# Three-Dimensional Physical Model in Urologic Cancer

**DOI:** 10.3389/fsurg.2022.757337

**Published:** 2022-05-25

**Authors:** Yu Xie, Guanlin Wu, Yu Liang, Gang Fan

**Affiliations:** ^1^Department of Urology, Hunan Cancer Hospital, The Affiliated Cancer Hospital of Xiangya School of Medicine, Central South University and the Clinical Research Center for Renal Tumor in Hunan Province, Changsha, China; ^2^The Clinical Research Center for Renal Tumor in Hunan Province, The Hunan Cancer Hospital and the Hunan Provincial Science and Technology Department, Central South University, Changsha, China; ^3^Department of Pathology, School of Basic Medical Sciences, Fudan University, Shanghai, China; ^4^Department of Urology, Huazhong University of Science and Technology Union Shenzhen Hospital, Shenzhen, China; ^5^The 6th Affiliated Hospital of Shenzhen University Health Science Center, Shenzhen, China

**Keywords:** three-dimensional printing (3D printing), urologic cancer, surgical planning and simulation, patient counseling, surgical education

## Abstract

Three-dimensional (3D) printing, as an evolving technology, enables the creation of patient-specific physical models with high precision; thus, it is widely used in various clinical practices, especially urologic cancer. There is an increasing need to clarify the contribution of 3D printing in the practice of urological cancer in order to identify various applications and improve understanding its benefits and challenges in clinical practice. Researches have focused on the use of 3D-printed models in patient and trainee education, surgical simulation, as well as surgical planning and guidance. This mini review will present the most recently published studies on the topic, including the applications of 3D-printed models, feasibility of performed procedures, possible simulated organs, application outcomes, and challenges involved in urologic cancer, to provide potential directions for future research.

## Introduction

3D printing technology, an emerging technology, is based on computer graphic data overlaying specific materials to create any shape of products. One of the most common printing methods is Fused Deposition Modeling (FDM)-that is, FDM builds structures by depositing material, like plastic, acrylonitrile butadiene styrene, polylactic acid, layer by layer (1). The technology bridges the gap between two-dimensional (2D) imaging and realistic anatomy, providing a more tangible method for the evaluation and comprehension of pathology (2). Thus, it has the potential to be increasingly important inpatient counseling (3), and in the training of residents and students (4, 5), as well as in surgical decision making and planning for complicated conditions and procedures (6).

This review aims to give an overview of the role of 3D-printed models currently used in urologic cancer, especially in renal cancer, bladder cancer, prostate cancer, and to explore future directions of research in this field.

## Methods

A systematic search was conducted in the electronic databases PubMed and Embase, and all relevant articles were published in English from their starting dates to June 2021 (Table 1). The search strategy used the following keywords relating to the 3D physical model printing (e.g., three-dimensional printing, 3D printing, 3D printed, 3D physical reconstruction, 3D model) in combination with urologic cancer (e.g. adrenal cancer, kidney cancer, urethral tumor, bladder cancer, prostate cancer, testicular cancer). All identified abstracts were carefully reviewed by two investigators (Y.X. and G.W.) independently for eligibility. The enrolled articles were excluded as follows: (i) 3D imaging reconstruction; (ii) 3D cell cultured system; and (iii) 3D printing in bioengineering. If the two investigators disagreed about the eligibility of an article, it was resolved by consensus with a third reviewer (G.F.). For the eligible articles included in this study, data were also extracted by two reviewers (G.W. and Y.L.), who reached a consensus on all of the data extraction items.

**Table 1:** Three-dimensional physical model in urologic cancer research.

**Urologic cancer**	**Applications**	**Procedure**	**Conclusions**	**References**
Renal cancer	Patient counseling	PN, LPN, RAPN, Cryoablation	Improve understanding of anatomy and the planned surgical procedure	(3, 16–19)
	Anatomical education	Anatomy, surgical processes	Improve understanding of anatomy	(5, 20)
	Surgical simulation	PN, LPN,RAPN	Can be as patient-specific surgical simulation tools; improve of surgical outcomes and robotic simulation education	(19, 21, 22)
	Surgical planning	PN, LPN, RAPN, Cryoablation	Reduction in estimated blood loss and warm ischemia time	(6, 8, 19, 22, 23)
	Surgical guidance	PN, LPN, RAPN, Cryoablation	Reduction in estimated blood loss and warm ischemia time	(6, 8, 23, 24)
Adrenal cancer	Surgical simulation	PA	Useful for understanding the patient's surgical anatomy	(25)
	Surgical planning	PA	Useful for planning the surgical procedures	(25, 26)
	Surgical guidance	PA	Useful for estimation of the remnant gland volume	(26)
Bladder cancer	Anatomical education	Anatomy, surgical processes	improved understanding of anatomy and surgical processes; improved student satisfaction with surgical training	(10)
	Surgical simulation	TURBT,UVA	Serve as a realistic platform for the medical training	(11, 27)
	Surgical outcome evaluation	Neobladder construction	Could be optional for surgical post-op evaluation	(13)
Prostate cancer	Patient counseling	RP, RARP, focal ablative therapy	improved understanding of anatomy, disease, tumor characteristics, and surgical procedure	(16, 18)
	Anatomical education	Prostate anatomy	Provided better orientation guide	(14)
	Surgical simulation	RP, RP (UVA), RARP, brachytherapy	Develop and build confidence in procedural skills	(22, 28–30)
	Surgical planning	RP, RARP, brachytherapy, Prostate biopsy	May improve prostatectomy outcome; educed missed detection in high-risk prostate cancer	(15, 31, 32)
	Surgical guidance	RARP	May improve prostatectomy outcome	(15)

*PN, partial nephrectomy; LPN, laparoscopic partial nephrectomy; RAPN, robot-assisted partial nephrectomy; PA, partial adrenalectomy; TURBT, transurethral resection of bladder tumor; UVA, ureterovesical anastomosis; RP, radical prostatectomy; RARP, robot-assisted radical prostatectomy; NS-RARP, nerve-sparing robot-assisted laparoscopic radical prostatectomy; VUA, vesicourethral anastomosis*.

## The Applications of 3D Printing in Clinical Practice

### Patient Counseling

Many patients have a limited understanding or even misunderstanding of their disease because of the limitation of anatomical knowledge and the complex anatomy. 3D models with both realistic anatomy and texture derived from imaging data could lead to a major paradigm shift in the way of patient-physician communication and patient counseling, thus improved patients' understanding of organ physiology, anatomy, tumor characteristics, and surgical procedure based on answers to questionnaires before and after being shown the3D-printed models, and result in supervisory treatment choices to patients (3).

### Anatomical and Surgical Education

**Figure 1:**
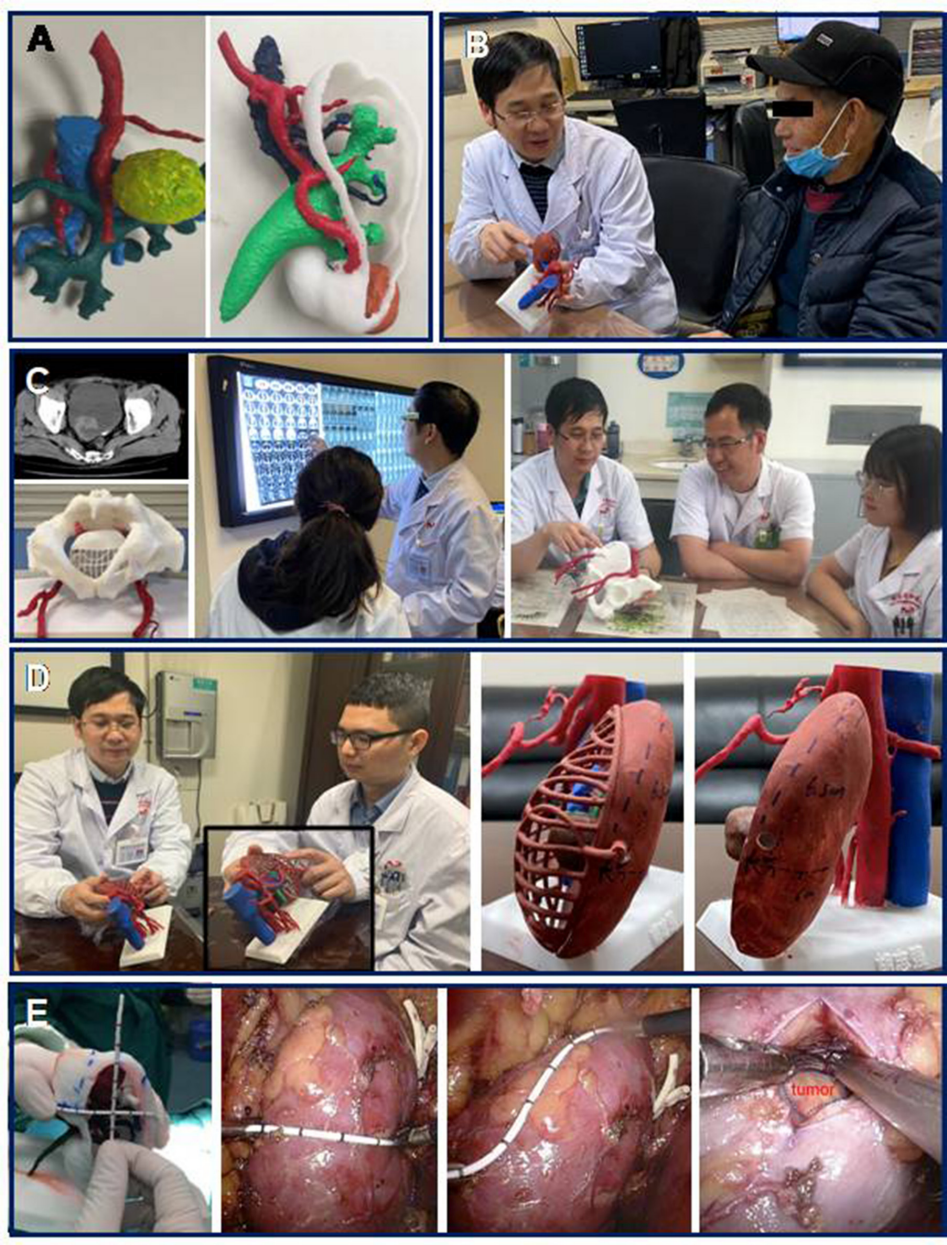
3D printing in clinical practices. 3D printing as an evolving technology enables the creation of patient-specific physical models with high precisionthat contribute to patient understanding, simulation training, as well as surgical planning and guidance. **(A)** 3D physical kidneymodels. **(B)** 3D-printed model in patient counseling. **(C)** 3D-printed model in anatomical and surgical education. Students were provided with 3D models during the training section. **(D)** 3D-printed model for preoperative planning. Surgeons discussed surgical planning and complexity, as well as potential complications, assisted by a patient-specific 3D model. Tumor markers are labeled on the surface of the models. **(E)** 3D-printed model for intraoperative guidance. 3D physical model utilisedin laparoscopic partial nephrectomy. Based on the makers on the surface of the kidney model, a renal mass can be quickly located with precise orientation.

Understanding anatomical pathology is critical and challenging to clinical education. 3D-printed models, which can reveal the anatomical relationships of organs and their surrounding tissues (Figure 1A), are intuitive and facilitate knowledge acquisition of anatomy, as well as surgical procedure-learning among medical students and junior trainees. Providing students/trainees with 3D physical models was found to be superior to all forms of 2Danatomy teaching (e.g., computed tomography (CT) and/or magnetic resonance imaging (MRI) and traditional anatomical text) assessed by anatomy test and subjective measures (4, 5).

### Surgical Simulation

3D-printed models have also been utilized as a simulator in partial nephrectomy, robot-assisted prostatectomy, transurethral resection of the prostate in both laparoscopic and robotic. The incorporation of 3D printing to develop surgical simulation models and training devices may prove to be a valuable resource to residents and faculty in surgical training exercises, that it allowed the trainees and junior clinicians to improve training quality and reduce learning curves (4, 7). Besides that, 3D models for a specific patient were presented to junior surgeons for preoperative surgical planning, contributing to understanding the surgical procedures, thus improving surgical performance and patient outcomes in our experience (6).

### Surgical Planning and Guidance

3D printing enables a 1:1 physical model, deriving from patients' individual imaging data on the basis of CT and/or MRI and has the potential to inform robotic, laparoscopic, and endoscopic systems for surgical planning in a more realistic manner than the review of 2D imaging alone. The accuracy of 3D-printed models was evaluated and discussed by surgeons and radiologists preoperatively and assisted to determine surgical approaches based on printed models. The surgical assistant adjusts the orientation of 3D models during operation to aid the surgeon in quickly and accurately locating the site of tumor masses based on the dimensions and landmarks on the printed models. Thus, it is widely used in preoperative surgical planning, primarily for minimally invasive and complex cases, such as partial nephrectomy and nerve-sparing radical prostatectomy. It has thus yielded encouraging applications in real-time surgical guidance that may help surgeons to improve both surgical precision and potentially, surgical results (6, 8).

## 3D Printing in Renal Cancer

Personalized 3D-printed models provide a novel way for patient-physician communication and preoperative counseling about complicated conditions and procedures (Figure 1B). 3D-printed kidneys provided to patients resulted in superior survey scores during the patient assessment of their satisfaction and understanding of their own pathology, as well as the surgical procedures, compared to review of 2D imaging alone (3).

The tactile “organ” can also help the students to better understand kidney physiology, anatomy, tumor characteristics, and surgical procedures. 3D-printed kidney-assisted contributes to the R.E.N.A.L. (Radius; Exophytic/Endophytic; Nearness; Anterior/Posterior; Location) nephrometry scoring assessment used by clinical students, when compared to traditional educational tools, reflecting the potential benefits of 3D printing in anatomical education (5).

In addition, 3D-printed kidneys have been utilized as simulators, primarily in partial nephrectomy (PN) simulation via robotic and laparoscopic systems, allowing trainees and junior clinicians the opportunity to improve training quality and reduce learning curves in both safety and effectiveness. The 3D kidney was presented to junior surgeons for preoperative simulation, contributing to their understanding of surgical procedures, and thus improving their surgical performance and patient outcomes (4).

Physical kidney models created by 3D printing can provide precise and individualized physical models that help surgeons to easily understand actual renal tumor characteristics, including tumor size, depth, and the relationship of location to the arteriovenous and collection systems, contributing to preoperative surgical programme determination and intraoperative guidance (Figure 1A) (6). 3D-printed models were welcomed during the performance of complex and high-precision surgeries (Figures 1D,E). The advantages of the 3D kidney for partial nephrectomy (PN) include shorter operation times and warm ischemia times, less intraoperative blood loss, as well as fewer complications, in comparison to PN with imaging alone (9). Our previous data revealed that, compared with traditional laparoscopic partial nephrectomy, 3D-printed models can help to reduce the warm ischemia time and the estimated intraoperative blood loss from 29.2 to 24.6 min, and 131.1 ml to 118.7 ml, respectively, in patients with complex renal tumor (R.E.N.A.L score>8) (8).

## 3D Printing in Bladder Cancer

Bladder radical cystectomy is a challenging procedure that medical students often experience difficulty with grasping the spatial characteristics of the pelvic region, involving pelvic organs, muscles, vessels, nerves, ligaments, and connective tissue over 2D imaging. 3D-printed models have been utilized in our center to facilitate the recognition of anatomical features within an educational setting and detected significant improvement in student satisfaction with training (Figure 1C). Importantly, the clinical education of students with 3D bladder models was superior to traditional educational methods, in terms of the students' understanding of the anatomy, treatment options, and surgical processes (10).

Another potential utilization of 3D models in bladder cancer is the simulation of transurethral resection of bladder tumor (TURBT). The TURBT model, which consisted of layers of mucosal, muscular, serosal, and perivesical fat, were simulated and demonstrated potential for incorporation of training and maintenance of advanced procedural skills (11). 3D reconstruction of blood supply to urinary bladder proved valuable for mock vascularized composite allograft bladder transplantation (12). Besides, the proposed application of 3D printing technology resides in the ability to evaluate novel Y pouch neobladder about structural and functional reconstructive outcomes (13).

## 3D Printing in Prostate Cancer

Similar to the applications of 3D printing in renal cancer, 3D prostate models would undoubtedly be useful for patient counseling, student education/training, as well as surgical planning and guidance. Compared to 2D imaging, augmented reality (AR) models and 3D computer models, 3D prostate physical models presented the greatest contribution to patient understanding of their diseases and prostate anatomy, tumor characteristics, as well as surgical procedures. 3D prostate models were also reported to be valuable for medical students but not for expert urologists in plotting tumor locations, since prostate MRI is arguably more difficult to interpret than other imaging (14).

The role of 3D models in assisting with prostate biopsy has the potential to enhance the histological correlation rate for prostate adenocarcinoma, with a detection rate of prostate cancer from 22.4% with systematic biopsies to 46.2% with targeted biopsies. Additionally, 3D-printed prostate models have shown great promise in surgical planning and guidance for tumor resection and may potentially aid in complex nerve-sparing radical prostatectomy, resulting in high accuracy of pathological margins and a promising approach (15).

## Conclusions

**Figure 2:**
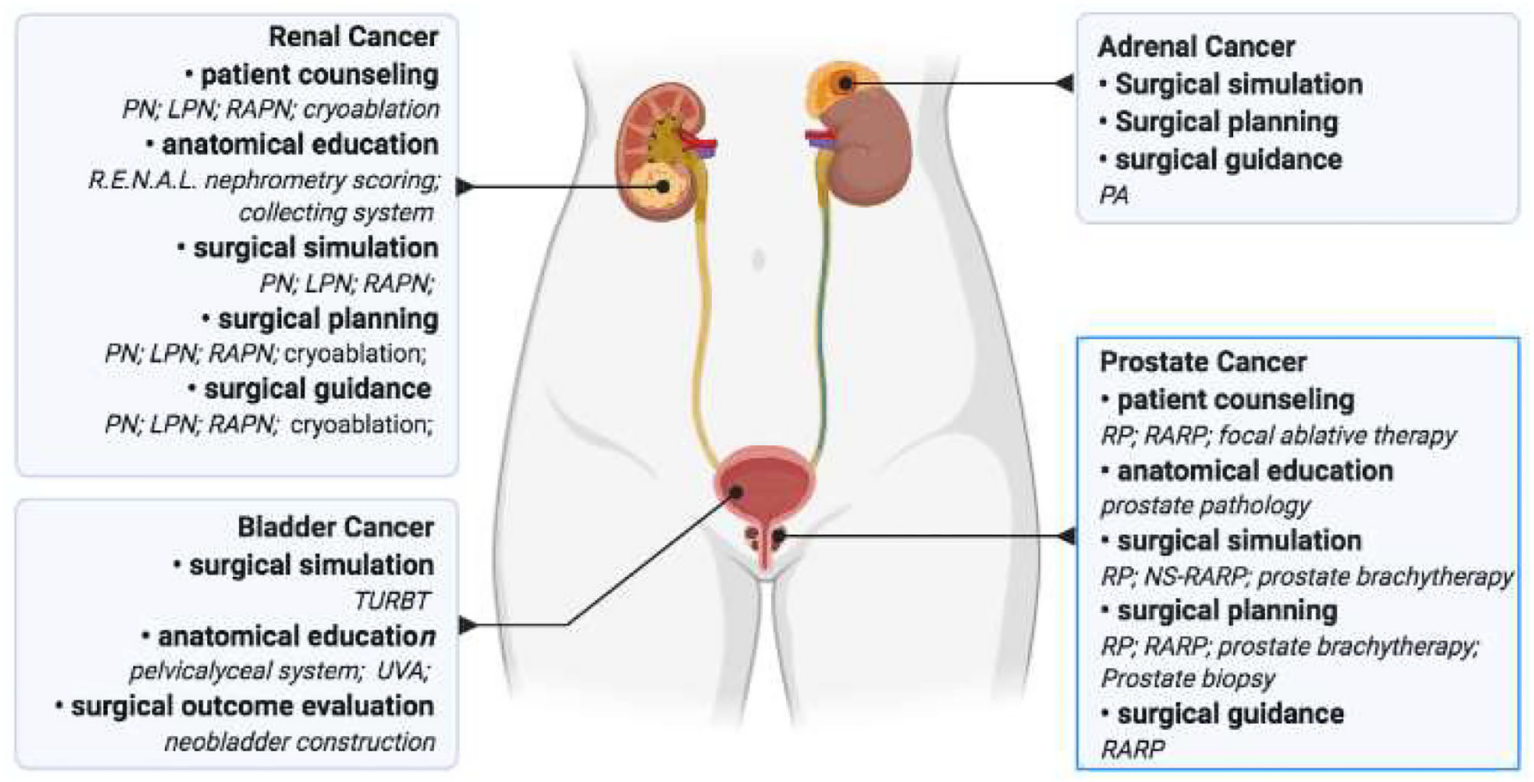
The application of 3D printing in urologic cancer. 3D printing was utilized in patient and traineeeducation, surgical simulation, as well as surgical planning and guidance in urologic cancer research. PN, partial nephrectomy; LPN, laparoscopic partial nephrectomy; RAPN, robot-assisted partial nephrectomy; PA, partial adrenalectomy; TURBT, transurethral resection of bladder tumor; UVA, uretherovesical anastomosis; RP, radical prostatectomy; RARP, robot-assisted radical prostatectomy; NS-RARP, nerve- sparing robot-assisted laparoscopic radical prostatectomy.

3D models assistant can overcome the challenges of complex anatomy and pre- and intraoperative sets required for clinical practices, thus allowing utilization in urologic cancer of kidney, bladder, as well as prostate. Apart from previously mentioned uses, proposed application of 3D printing technology resides in the ability to utilized insurgical planning and guidance of adrenal part resection and testicular sparing surgery (Figure 2). With improvements in technology and materials, cost which derived from the initial purchase of the printer and printing materials will result in lower production and equipment costs (2). However, the level of quality evidence on 3D technology reported in the last decade is not high enough. Large-scale evidence and comparative trials are required to elucidate 3D printing's benefits to clinical practices in urology.

## Author Contributions

YX and GF drafted the article. All authors contributed to its completion.

## Funding

This study was funded by the clinical research center for renal tumor in Hunan province (2020SK4006).

## Conflict of Interest

The authors declare that the research was conducted in the absence of any commercial or financial relationships that could be construed as a potential conflict of interest.

## Publisher's Note

All claims expressed in this article are solely those of the authors and do not necessarily represent those of their affiliated organizations, or those of the publisher, the editors and the reviewers. Any product that may be evaluated in this article, or claim that may be made by its manufacturer, is not guaranteed or endorsed by the publisher.
